# Enhancement of Osteogenic Differentiation and Proliferation in Human Mesenchymal Stem Cells by a Modified Low Intensity Ultrasound Stimulation under Simulated Microgravity

**DOI:** 10.1371/journal.pone.0073914

**Published:** 2013-09-12

**Authors:** Sardar M. Z. Uddin, Yi-Xian Qin

**Affiliations:** Orthopaedic Bioengineering Research Laboratory, Department of Biomedical Engineering, Stony Brook University, Stony Brook, New York, United States of America; University of California, San Diego, United States of America

## Abstract

Adult stem cells can differentiate into multiple lineages depending on their exposure to differing biochemical and biomechanical inductive factors. Lack of mechanical signals due to disuse can inhibit osteogenesis and induce adipogenesis of mesenchymal stem cells (MSCs). Long-term bed rest due to both brain/spinal cord injury and space travel can lead to disuse osteoporosis that is in part caused by a reduced number of osteoblasts. Thus, it is essential to provide proper mechanical stimulation for cellular viability and osteogenesis, particularly under disuse conditions. The objective of this study was to examine the effects of low intensity pulsed ultrasound (LIPUS) on the osteogenic differentiation of adipose-derived human stem cells (Ad-hMSC) in simulated microgravity conditions. Cells were cultured in a 1D clinostat to simulate microgravity (SMG) and treated with LIPUS at 30mW/cm^2^ for 20 min/day. It was hypothesized that the application of LIPUS to SMG cultures would restore osteogenesis in Ad-hMSCs. The results showed significant increases in ALP, OSX, RANKL, RUNX2, and decreases in OPG in LIPUS treated SMG cultures of Ad-MSC compared to non-treated cultures. LIPUS also restored OSX, RUNX2 and RANKL expression in osteoblast cells. SMG significantly reduced ALP positive cells by 70% (p<0.01) and ALP activity by 22% (p<0.01), while LIPUS treatment restored ALP positive cell number and activity to equivalence with normal gravity controls. Extracellular matrix collagen and mineralization was assessed by Sirius red and Alizarin red staining, respectively. SMG cultures showed little or no collagen or mineralization, but LIPUS treatment restored collagen content to 50% (p<0.001) and mineralization by 45% (p<0.001) in LIPUS treated-SMG cultures relative to SMG-only cultures. The data suggest that LIPUS treatment can restore normal osteogenic differentiation of MSCs from disuse by daily short duration stimulation.

## Introduction

Adult mesenchymal stem cells (MSCs) are multi-potent stem cells capable of self-renewal and differentiation into osteoblastic, adipogenic, myogenic, and chondrogenic lineages. Recent studies have examined the effects of the presence or absence of mechanical stimuli on commitment of MSCs to different lineages [1,2,3]. Mechanical vibrations, stress, and shear forces can all enhance osteogenic differentiation of MSCs while the lack of mechanical stimuli can induce adipogenesis [2,4]. Luu et al. have shown that low magnitude mechanical stimulation (LMMS) induces osteogenesis and inhibit adipogenesis [5,6]. MSCs extracted from LMMS treated mice showed increased expression of RUNX2 relative to PPARγ [5]. A recent study by Yang et al. has shown that cyclic tensile strain increased RUNX2 expression while inhibiting PPARγ in rat MSCs [7]. In contrast, lack of mechanical stress can induce adipogenesis and inhibit osteogenesis [8,9]. Loss of gravity in space or lack of physical activity due to spinal/brain injury can significantly reduce mechanical stresses thus leading to decreased rates of osteogenesis and increased rates of adipogenesis [6]. The lack of mechanical stress during spaceflight induces bone loss of 1-2% per month, which can eventually lead to disuse osteoporosis [10].

MSCs are progenitor cells to osteoblasts, and the detrimental effects of disuse on MSCs can significantly reduce their rates of proliferation and differentiation into osteoblasts [8]. Microarray analysis of MSC cultures in simulated microgravity (SMG) have shown reductions in expression of genes controlling the cell cycle, cytoskeleton, proliferation, and differentiation along with increased expression of apoptotic genes [9]. Furthermore, MSCs cultured in SMG had disorganized microfilaments and reduced F-actin polymerization [8,11]. Huang et al. have shown that this is in part due to inhibition of the ERK1/2 and AKT pathways, leading to reductions in proliferation, RUNX2, and ALP concomitant with increased PPARγ levels [11]. In general, lack of mechanical stimulus appears to force MSCs into a state of quiescence.

To restore MSCs self-renewal and osteogenic differentiation, it is essential to provide an anabolic mechanical signal, ideally one that provides a localized mechanical stimulus. LIPUS produces pressure waves that induce biochemical events in bone cells [12,13,14]. The effects of LIPUS on intercellular activity, cytokine release [15], gene expression [16], calcium mineralization [17], Akt signaling [18], potassium influx [19], angiogenesis [20], adenyl cyclase activity, and TGF-b synthesis [21] have all been studied.

LIPUS induced mechanical deformations activate receptors on cell membranes such as integrins, mechanosensitive-calcium channels, G-proteins, IGF, TGF-β/BMP and gap junctions, activating different downstream pathways [18,22,23,24,25,26]. These studies, as well as many others, show that rates of bone formation are increased in the presence of LIPUS.

Several studies have specifically evaluated the effects of ultrasound on mechanotransductive pathways. Tang et al. reported on activation of the Akt pathway and P-13-kinase, through aggregation of integrin expression, which resulted in induction of nitric oxide, hypoxia inducible factor-1, and increased activity of COX-2 in osteoblast cells [24,25]. Similarly, LIPUS treated osteoblasts show higher nuclear localization of β-catenin and activation of Wnt signaling [27]. A microarray study done on LIPUS-treated osteoblasts showed enhanced gene expression of integrins, cytoskeletal components, TGF-β family members, IGF family members, MAPKs pathway, ATP-related, Guanine nucleotide binding protein family, lysyl genes, and apoptosis-associated gene families as compared to non-treated osteoblast [28,29,30,31]. These cellular level studies indicate that ultrasound treatment can activate mechanotransductive pathways and significantly increase osteogenic differentiation in progenitor cells and promote osteogenic maturity in differentiated osteoblasts. Recent studies have also shown increased RUNX2, ALP, Collagen type 1, and integrin beta 1 expression in LIPUS treated MSCs [32]. Finally LIPUS exposure has been shown to enhance cell adhesion, focal adhesion formation, and increase proliferation in MSCs [32].

The catabolic effects of disuse are driven by inactivation of mechanotransductive pathways, leading to reduced MSC activity. The mechanotransductive properties of LIPUS have the potential to induce mechanical stress in MSCs and promote osteogenic differentiation in microgravity or disuse conditions. The objective of this study was to evaluate the hypothesis that LIPUS exposure restores the osteogenic differentiation of SMG cultured MSCs under simulated cellular microgravity.

## Materials and Methods

### Rotation Microgravity Simulation

**Figure 1:**
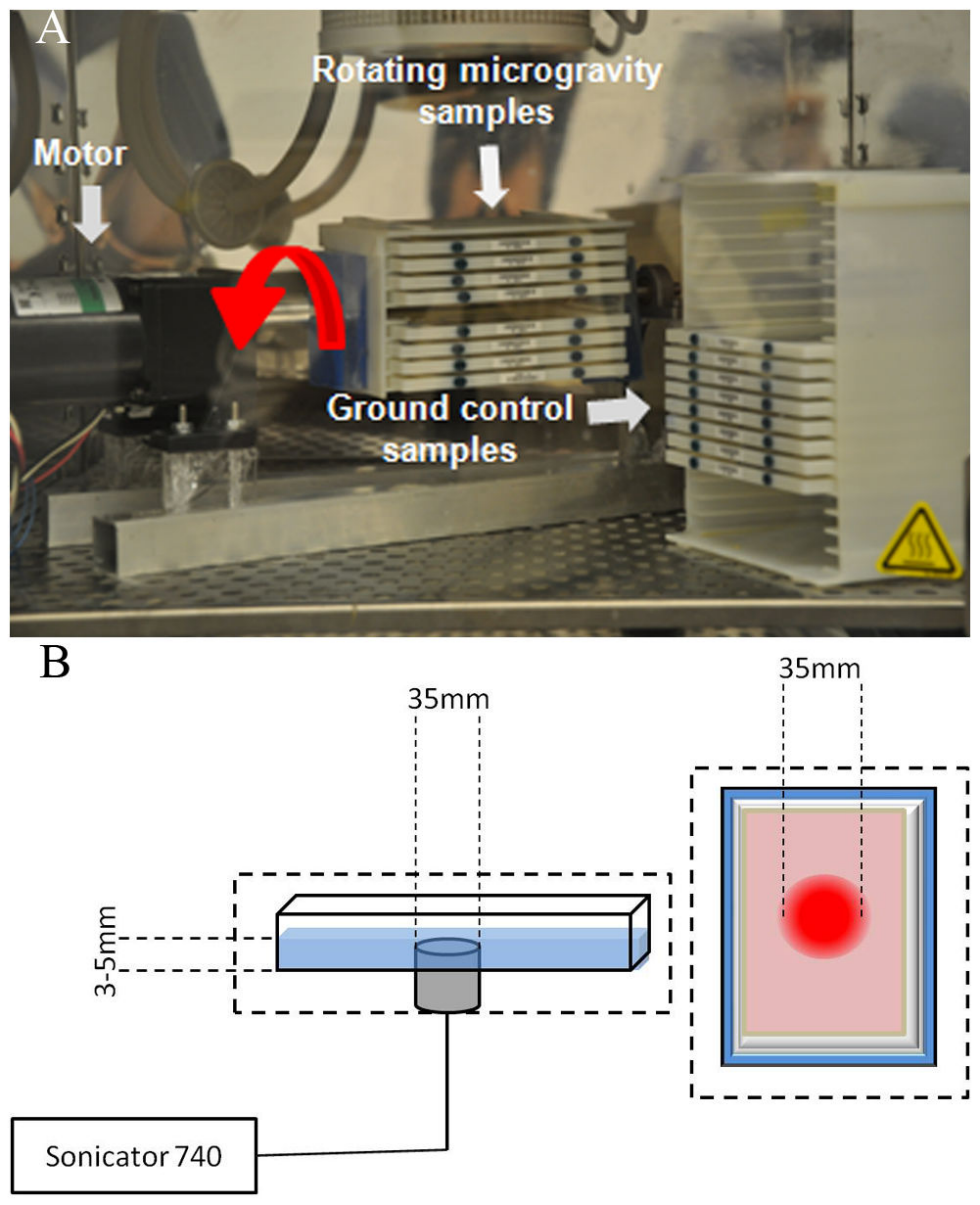
Simulated microgravity (SMG) bioreactor. a) One dimensional rotation results in net gravity vector of magnitude zero, thus producing simulated microgravity. SMG bioreactor setup inside incubator with experimental and control samples setup b) Ultrasound setup schematics showing samples positioning relative to ultrasound probe. Samples were kept inside incubator during ultrasound exposure.

Our lab developed a *one-dimensional clinostat* (*1-D Clinostat*) to keep cells in continuous 1-D rotation at a speed of 15 rpm around the horizontal axis, averaging out the net gravitational forces (Figure 1) [33]. Lee Silver et al. explained in detail the effects of 1-D rotation on cells and sub-cellular organelles with respect to gravity, in which the authors demonstrated how continuous rotation along the horizontal axis both disables cells’ ability to respond to gravitational forces and induces a lack of orientation [34]. A variable speed motor controls the rotator. The holder is 80 mm (radius = 40mm) in width, allowing it to hold eight Opticell® (Nunc, VWR, Bridgeport, NJ, USA) cartridges, resulting in a radial force of less than 0.045 N.

Each Opticell cartridge is 2 mm thick and MSCs were seeded on their gas-permeable polystyrene membranes. Each cartridge was filled with 10 ml of media and all air bubbles were carefully removed to prevent fluid shear stress. The clinostat was kept in sterile conditions at 37°C in 5% CO_2_ in a standard humidified incubator (Thermo scientific, Asheville, NC, USA) for the duration of all experiments.

### Ultrasound Exposure

Sonicator® 740 (Mettler Electronics, Anaheim, CA) with a 10 cm^2^ transducer (35 mm diameter) was used for LIPUS stimulation, utilizing a modified repetitive frequency at 100 Hz pulse, an ultrasound characteristic frequency of 1 MHz and a pulse width of 200 μs repeated at 100 Hz at an intensity of 30 mW/cm^2^ for 20 minutes/day(I_SATA_). The region of LIPUS treatment was marked with a 10 cm diameter circle before the start of each experiment. All stimulations targeted the same region of each Opticell (each Opticell cartilage was stimulated individually), and only those cells that were growing in the Region of Stimulation (RoS) were harvested and analyzed. The detail schematics of Ultrasound setup have been shown in Figure 1b. All the stimulations were done inside an incubator in sterile conditions. The 3-5mm distance between the transducer and Opticell was filled with degassed water.

### Cell Culture

Adipose derived human Mesenchymal stem cells (Ad-hMSC, LifeLine, CA) were cultured in StemLife media (LifeLine, CA) at an initial seeding density of 250,000 cells/Opticell. Cells were distributed into five groups (n=4/group): 1) Control; 2) Gravity (G); 3) Gravity + LIPUS (GU); 4) SMG (M); and 5) SMG + LIPUS (MU). All the groups were initially cultured in proliferation media (StemLife media) until 90% confluency was reached. After 90%, GU, M, and MU groups were supplemented with osteogenic factors (50 ug/ml ascorbic acid, 10 mM beta-glycerolphosphate), while the control group was maintained in proliferation media to serve as a negative control for osteogenesis. Microgravity simulation of M and MU groups was started after initiation of osteogenic induction. GU and MU samples were stimulated with LIPUS for 20 min per day, 5 days a week, for the duration of the experiments while the G and M samples were placed on a switched-off transducer for the same duration.

Human fetal Osteoblasts (Hfob 1.19, ATCC, Manassas, VA.USA) were cultured in DMEM:F12 media supplemented with 15% FBS, 0.3 mg/ml G418 with an initial seeding density of 250,000 cells/Opticell. Cells were distributed into four groups (n=4/group): 1) Gravity (G); 2) Gravity + LIPUS (GU); 3) SMG (M); and 4) M + LIPUS (MU). GU and MU samples were stimulated with LIPUS for 20 min/day for the duration of the experiments while G and M samples were placed on a switched-off transducer. All cells were maintained at 37°C and 5% CO_2,_ and the culture medium was changed every other day.

### Quantitative Real-Time PCR

After 24 hours of SMG, MU and GU samples were exposed to LIPUS and immediately lysed with lysing buffer for RNA collection. The RNA from the control, G, and M was collected in similar fashion. Total RNA was extracted using RNeasy Mini Kit (Qiagen) and reverse transcribed using random primers and a High capacity RNA-to-cDNA kit (Applied Biosystems). The cDNA was then amplified using a StepOnePlus Real-time PCR system (Applied Biosystems). QPCR analyses were then performed for ALP, RUNX2, OSX, *RANKL* and OPG Taqman primers purchased from Applied Biosystems.

Hfob cells were treated with LIPUS in similar manner to study the comparative expression in RUNX2, ALP, RANKL and OPG. All the data was normalized to GAPDH expression to study relative expression of the targeted genes.

### ALP Activity

After 7 days of SMG, samples from all groups were washed twice with double distilled water and lysed using sonification. Cell lysates were incubated with p-nitrophenol phosphate (Sigma) at 37°C for 1 hr. The enzymatic reaction was stopped using 1 M sodium hydroxide and absorbance was measured at 540nm (Bio-Tek EL800, Winooski, VT, USA)

### Fluorescence-activated cell sorting – FACS

Ad-HMSCs were trypsinized, washed with PBS, and stained with primary anti-bone alkaline phosphatase (abcam, ab17272) and secondary goat-anti mouse lgG1 heavy chain (FITC) antibodies (abcam, ab97239). After staining, the cells were washed, fixed with 1% formalin, and analyzed for ALP positive cells using flow cytometry.

### Collagen Staining

After 12 days of treatment, the RoS were cut out of the Opticells and fixed in 4% paraformaldehyde for 30 min at room temperature. The samples were then stained with 1% pico-sirius red for 1 hr and the cells were washed with acidified water (0.5% acetic acid water) and dehydrated with serial ethanol washes: 70%, 90%, and 100% (5 min each). Digital images were taken using polarized light microscopy (Nikon Diaphot 200, Melville, NY, USA) at 2.5X magnification. After imaging, staining was eluted using sodium hydroxide and quantified at 540nm optical density. 

### Matrix Mineralization

After 12 days of treatment, the RoS were cut out of the Opticells and the cells were fixed in 70% ethanol for 1 hr at room temperature. They were stained with 40mM alizarin red (pH 4.2) for 10 min, washed with tap water, allowed to dry at room temperature and imaged using an Axiovert 2000M Inverted Microscope (Carl Zeiss, Axiocam MRC, Thornwood, NY). Following imaging, the stain was eluted off the cells in a 10% cetylpyridinium chloride 10mM sodium phosphate solution for 15 min. The eluted stain was then measured at 562nm in a spectrophotometer (Bio-Tek EL800, Winooski, VT, USA). All samples were quantified against an alizarin red standard curve in 10% cetylpyridinium and normalized to the total number of cells.

### Statistics

The GraphPad Prism 3.0 software was used to run statistical analyses. All of the data is presented in average ± standard deviation format. One-way ANOVA with Newman Keuls Post -hoc was used to calculate significance within the different groups and time points. Significant p-values were considered to be <0.05.

## Results

### LIPUS increases expression of osteogenic genes in Ad-HMSC

**Figure 2:**
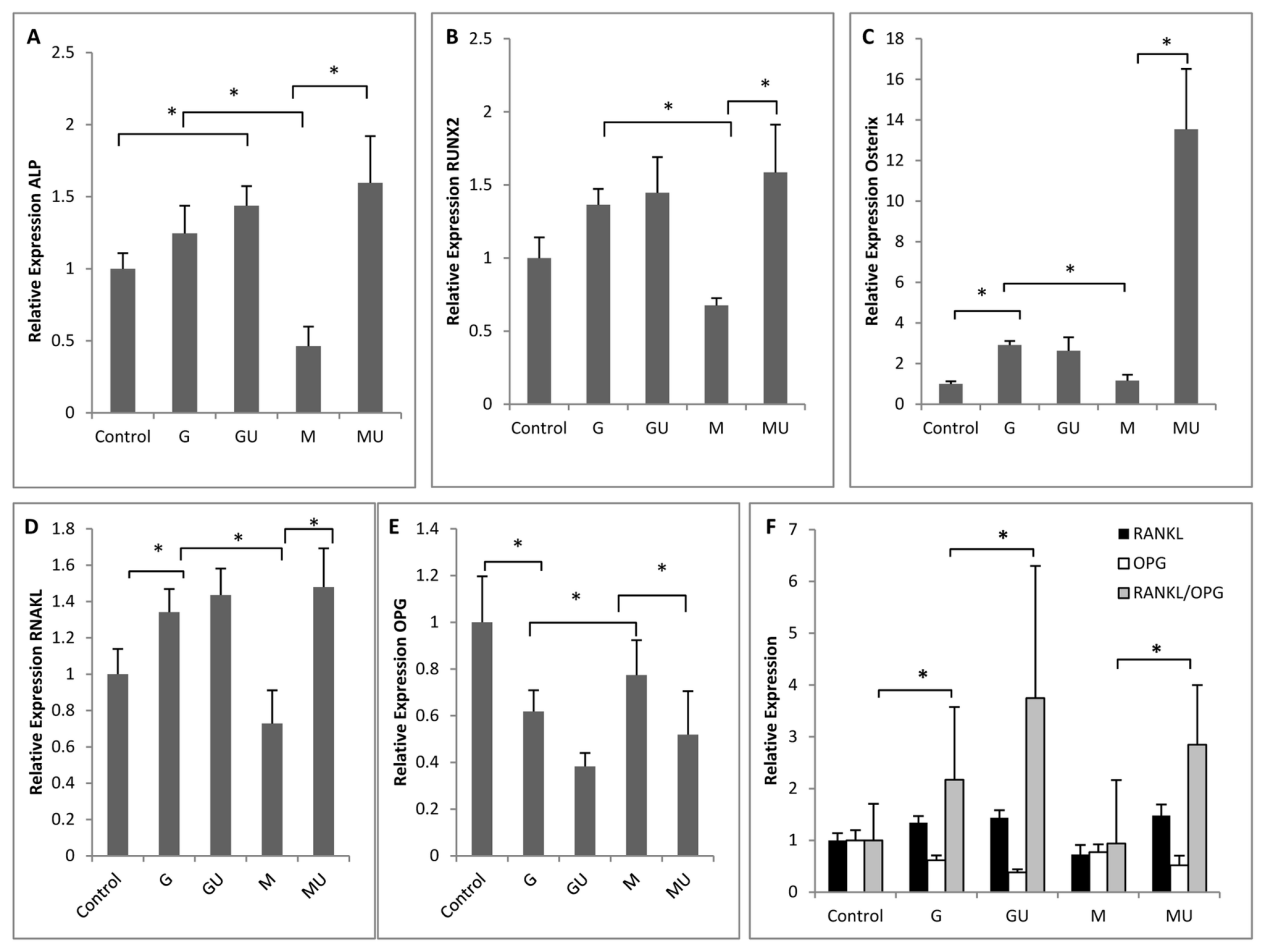
PCR analyses of osteogenic genes in response to SMG and LIPUS exposure in hMSC. a) ALP expression decreases in SMG and application of LIPUS significantly increases ALP expression; highest expression observed in MU samples. b) OSX gene expression significantly reduces in SMG and LIPUS stimulation significantly increase in OSX in MU samples relative to M samples. LIPUS doesn’t induce any significant change in GU samples relative to G samples. c) RUNX2 expression. MU samples show significant increase compared to M samples and LIPUS exposure restores RUNX2 expression in HMSC. d) RANKL expression levels significantly reduce in M samples in SMG environment. LIPUS exposure significantly increases RANL expression. RANKL expression in GU samples shoe little to no change relative to G samples) M samples showed significantly higher OPG expression than G, GU and MU samples. Exposure of LIPUS further reduces OPG expression ad-HMSC. f) RANKL/OPG ratio is indicative of osteogenic commitment: M samples show same ratio as Controls and LIPUS exposure significantly increased RANKL/OPG ratio. (* p < 0.05)

PCR analyses were completed to assess the effects of SMG and LIPUS on the expression of osteogenic genes. SMG significantly reduced expression of all the genes analyzed (ALP, RUNX2, OSX, RANKL, and OPG) (Figure 2). Application of LIPUS increased gene expression of ALP, RUNX2, OSX, RANKL (Figure 2a-d), and reduced expression of OPG (Figure 2e). MU samples showed significant increases in expression of ALP, RUNX2, OSX and RANKL relative to M samples, and restored the expression of these genes to levels observed in G samples (Figure 2a-d). In the GU samples, LIPUS exposure showed positive correlations with osteogenic gene expression but no significant differences were found relative to G cultures. OPG expression levels reduced significantly with application of LIPUS in the GU and MU samples (Figure 2e). When combined with RANKL expression, an increased RANKL/OPG ratio was seen following application of LIPUS, with the highest ratio seen in GU and MU samples. This finding suggests that LIPUS stimulation favors bone formation over bone resorption (Figure 2f).

Our data from osteoblast gene expression of OSX, RUNX2 and RANKL confirms the anabolic role of LIPUS in osteoblast cells. RUNX2 and OSX expression in Hfob showed down-regulation after 24 hours of SMG. MU samples showed significant increases in RUNX2 and OSX immediately after LIPUS stimulation. GU samples showed little to no increase in RUNX2 and OSX expression levels after LIPUS stimulation.

**Figure 3:**
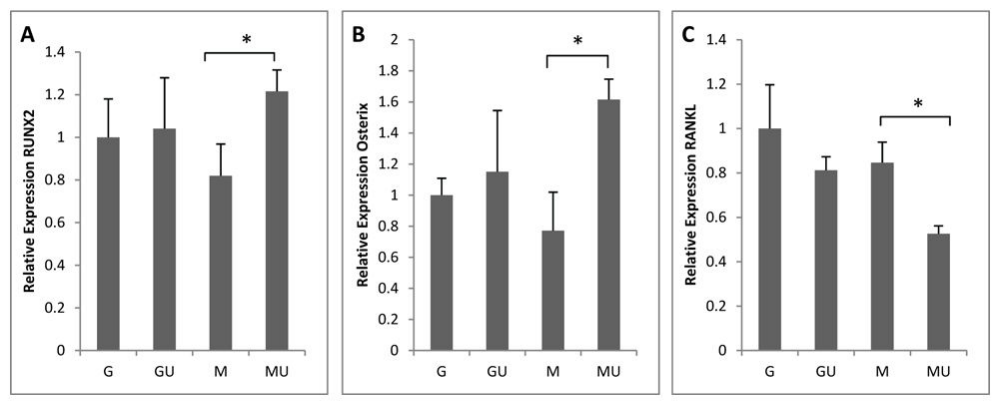
LIPUS increases osteogenic genes expression and reduces osteoclastogenic gene expression in Hfob cells. a) RUNX2 showed no change in GU but was significantly reduced in M cultures. Application of LIPUS increased RUNX2 expression significantly relative to M cultures. b) OSX decreased significantly in M cultures and application of LIPUS increased OSX expression significantly in MU but didn’t show any significant changes in Gravity samples. c) RANKL expression showed decreases M; exposure of LIPUS further decreased RANKL expression in MU cultures contrary to LIPUS effect on hMSC (Figure 2c). LIPUS exposure show little change in RANKL expression in GU samples.

RANKL expression was studied to see the effects of LIPUS on osteoclastogenesis when exposed to SMG conditions. RANKL expression showed little or no change due to SMG, however LIPUS exposure significantly decreased RANKL expression (Figure 3, p<0.05) in MU cultures relative to G and M.

### LIPUS restores ALP positive population and ALP activity in Ad-HMSC

**Figure 4:**
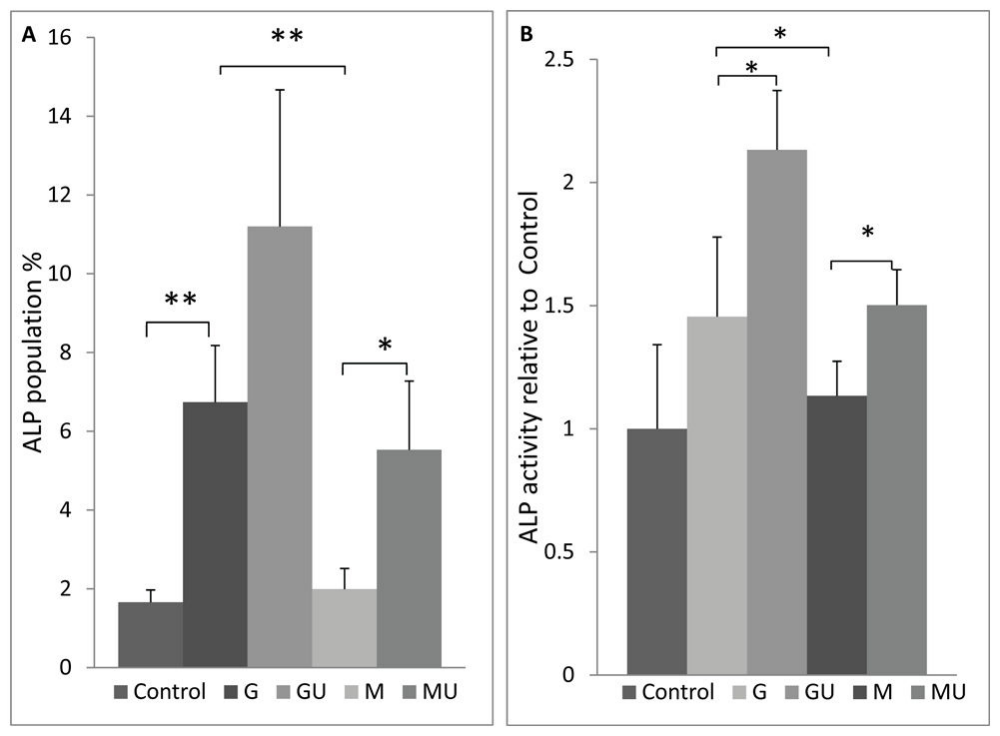
ALP positive population and activity. a) ALP positive population signigicantly goes down to levels of control in M cultures. Application of LIPUS restores the ALP positive population in MU samples and significantly increases in GU samples relative to G samples. b) ALP Activity showed same pattern as ALP positive population, as ALP activity in M samples was significantly reduced and application of LIPUS restored ALP activity in differentiating cells. Application of LIPUS increases ALP activity in GU cultures. (*p<0.01, ** p< 0.001).

ALP, a membrane bound enzyme is considered to be the initial marker of osteogenesis as MSCs differentiate into osteoblasts. We looked at both the percentage of ALP positive cells and total ALP activity levels in ad-MSC under SMG with and without LIPUS exposure. SMG reduced the ALP positive population in M group, 70% less than G, resulting in levels similar to control (Figure 4). Exposure of LIPUS significantly increased the percentage of ALP positive cells in GU samples by 3% and restored ALP positive cells in MU samples in comparison to gravity controls (G).

ALP positive cells and ALP activity were significantly reduced in M cultures relative to gravity controls (G) (Figure 4b). However, LIPUS stimulation increased ALP activity in GU samples by 45% and completely restored ALP activity in MU samples relative to G samples (Figure 4b).

### LIPUS exposure significantly reverses SMG-induced decreases in collagen production

**Figure 5:**
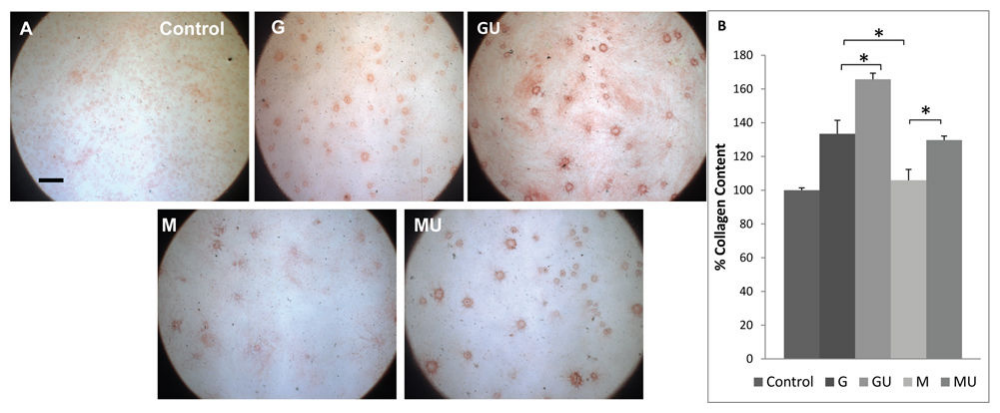
Collagen Content and Quantification. a) Sirius Red stain showing collagen clusters forming in extracellular matrix. Control samples show little or no collagen and Gravity (G) samples showed evenly distributed collagen clusters over the ECM. Simulated microgravity (M) samples showed little or no collagen clusters and much more comparable to control cultures. Application of LIPUS to simulated microgravity + LIPUS (MU) samples apparently increased collagen content, making them more comparable to G samples. Gravity + LIPUS (GU) samples showed denser collagen clusters and formation of thick collagen fibers (scale bar 500um). b) Sirius red quantification showed a significant decrease in collagen content in M cultures to the levels of control. Application of LIPUS restores collagen content in MU cultures, making them more comparable to G samples. LIPUS exposure significantly increases collagen in MU and GU samples (*p < 0.001).

Collagen deposited by osteoblasts was measured using Sirius red (stains collagen, predominantly type I and III fibers). Histological/qualitative analyses showed a reduction in collagen level in the SMG exposed samples (M) and significant recovery due to stimulation with LIPUS (MU), as evidenced by dark red clusters of collagen evenly distributed throughout the RoS (Figure 5a). The application of LIPUS on the GU samples resulted in a significant increase in collagen secretion as compared to the control (G) (Figure 5a). In addition, while all three cultures, G, GU and MU showed variable collagen density (with scattered areas of lower and denser clusters of collagen), the M cultures showed weaker staining and overall low collagen density throughout the cultures (Figure 5a). Further, quantitative measurement of the intensity of Sirius Red staining in these cultures identified a significant reduction of 21% of collagen secretion in the presence of SMG in comparison to the G samples (Figure 5b). In contrast, LIPUS stimulation reversed this trend and restored collagen levels to those observed in the G samples and enhanced collagen content in GU samples by 24% (Figure 5b).

### LIPUS exposure significantly reverses SMG-induced decreases in calcification

**Figure 6:**
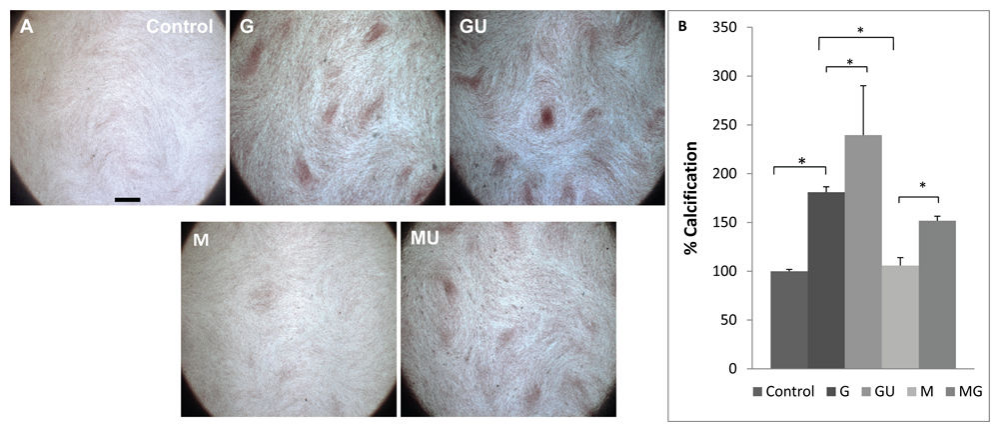
Matrix mineralization and Alizarin Red Quantification. a) No or little mineralization was apparent in Control samples. Gravity samples showed formation of mineralized clusters distributed evenly over the area of ECM. M samples showed no visible clusters and ECM morphology comparative to Controls. Application of LIPUS in MU samples induced mineralization as calcium clusters are apparent in ECM and morphology was more comparable to G samples. GU cultures showed denser calcium cultures and with higher frequency (scale bare 500um). b) Alizarin red quantification showed significant decrease of ECM calcification in M cultures almost to the level of the control group. LIPUS exposure restored mineralization of ECM. Application of LIPUS significantly increased calcification in MU cultures relative to M and GU cultures relative to G. (*p<0.001).

To further probe the positive effects of LIPUS stimulation on SMG-exposed cells, matrix calcification was investigated using Alizarin Red staining. Results from these experiments showed even distribution of calcium phosphate in G cultures; however, with LIPUS stimulation, a much denser and wider pattern of calcification was observed in ECM (Figure 6a, GU). M cultures showed decreased levels of Alizarin Red staining comparable to the control samples (Figure 6a, M). LIPUS exposure in SMG (MU) samples showed increased distribution of calcium phosphate nodules resembling that seen with G (Figure 6a, G). LIPUS exposure also enhanced alizarin red staining in GU cultures, leading to the formation of denser positively stained clusters. Quantification of Alizarin red stain showed a 75% decrease in matrix calcification in M compared to G, but with LIPUS stimulation, the stain showed a 45% increased calcification in the MU cultures. Furthermore, there was a significant 56% increase in calcification in the GU cultures in comparison to G (Figure 6b).

## Discussion

Recent studies have shown that both mechanical and biochemical stimuli are important for differentiation of stem cells into different lineages. Lack of mechanical stress significantly reduces a MSC’s capability to differentiate into an osteoblast, which may lead to disuse osteoporosis. Furthermore, Yamazaki et al. showed that a lack of mechanical stress can inhibit osteogenesis and induce adipogenesis in bone marrow MSCs [8]. MSCs play an important role in bone remodeling, as they are the progenitor cells to osteoblast cells, and also affecting osteoclastogenesis through regulation of OPG. The study was designed to evaluate the effects of LIPUS as a potential countermeasure to MG induced inhibition of osteogenic differentiation of MSCs. LIPUS is an acoustic pressure wave capable of providing localized mechanical stimulation that leads to activation of cell membrane bounded stretch receptors, ion channels, G-protein coupled receptors and integrins. The preliminary results have demonstrated that acoustic force can serve as a form of mechanical signals to trigger cell osteogenesis. In the presence of biochemical and mechanical stimuli, MSCs differentiate into bone matrix secreting osteoblasts, characterized by elevated expression of ALP, Runx2, OSX, and RANKL, along with secretion of matrix proteins. The present study confirmed the inhibitory effects of SMG on osteogenic differentiation of MSCs; SMG cultures showed reduced expression of ALP, RUNX2, OSX, RANKL and increases in OPG. In addition, SMG-MSCs showed fewer ALP positive cells and reduced ALP activity, which was more comparable to the non-induced MSCs (control). SMG-MSCs also showed little or no collagen production or matrix mineralization after 12 days of culture in osteogenic media. The data show that SMG-MSC (M) are drastically hampered from osteogenesis after 12 days of culturing in osteogenic media and instead resemble non-induced MSC cultures. These results confirm that lack of gravity or mechanical stress severely affects MSC osteogenic differentiation. Meyers et al. have reported similar results with ALP and RUNX2 expression and collagen content under SMG [35]. The same study reported inactivation of the MAPK-ERK pathway due to reduced expression of collagen 1 in extracellular matrix leading to inhibition of RUNX2 expression and osteogenesis [35]. Furthermore, Sheyn et al. have shown that SMG down-regulates genes regulating cell proliferation, differentiation, adhesion, cytoskeletal proteins, and cell communication and upregulates stress-related genes in human MSCs [36].

LIPUS exposure increased expression of ALP, OSX, RUNX2, and RANKL and decreased OPG gene expression in SMG cultures. LIPUS restored OPG gene expression to levels similar to normal in gravity grown control samples. RANKL and OPG are proteins secreted by osteoblasts and MSCs to control osteoclastogenesis. As MSCs differentiate into osteoblasts, the level of RANKL increases while that of OPG decreases. LIPUS exposure increased the RANKL/OPG ratio in normal and SMG cultures, thus enhancing osteogenic differentiation of MSCs. Similarly, recent studies by Liu et al. have shown that static and dynamic pressure can increase the RANKL/OPG ratio in early osteogenesis [37]. These findings correlate well with our study, as LIPUS is an acoustic pressure wave providing localized and targeted stimulation. On the contrary, Rubin et al. has reported that cyclic strain reduces RANKL expression in murine marrow stem cells [38]. It is speculated that different mechanical stimuli can induce different responses in MSCs. Detailed study of mechanical stimuli relative to corresponding mechanotransductive pathways is required to understand MSC role in osteoclastogenesis and osteogenesis. The osteoblast RUNX, OSX and RANKL expression data confirmed inhibitory effects of SMG on osteogenic gene expression. LIPUS exposure restored RUNX2 and OSX expression in osteoblast but further reduced expression of RANKL, RUNX2 and OSX are established osteogenic markers. Increased levels of RUNX2 and OSX indicate anabolic effects due to LIPUS treatment. Conversely, RANKL is known to induce osteoclastogenesis and osteoclast activity, thus the reduced RANKL expression seen in LIPUS treated groups (MU & GU) is indicative of the anti-resorptive effects of LIPUS stimulation in osteoblast cultures. Gene expression analysis, along with recently published data from our lab showing enhanced mineralization in osteoblast, shows LIPUS potential as an anabolic and anti-resorptive stimulus for osteoblast cells [33]. Together, gene expression data from MSC and osteoblast show inhibitory effects of SMG on osteogenesis and possible increase in osteoclastogenesis. LIPUS stimulation in SMG conditions significantly increases the expression of RUNX2, ALP, and OSX while reducing RANKL expression, indicating increased osteogenesis but reduced osteoclastogenesis.

ALP positive population and activity increased with exposure to LIPUS stimulation. SMG cultures had little or no ALP positive cells; application of LIPUS restored ALP positive cells. ALP is a membrane bound enzyme that functions to catalyze phosphate addition to calcium ions resulting in the formation of hydroxyapatite crystals and matrix mineralization. Since ALP expression is known as an early osteogenic marker, increases in ALP positive cell numbers and ALP activity is indicative of MSC osteogenic differentiation, as seen in the LIPUS treated cultures.

Collagen and matrix mineralization also increased with the application of LIPUS. Collagen is an extracellular matrix protein that facilitates cellular adhesion, mineralization, and mechanotransduction through integrin α2β1 [35]. The reduction of collagen content inhibits integrin α2β1 activation, formation of focal adhesion complexes and MAP-ERK pathways, which are essential from osteogenic induction. LIPUS exposure restores collagen in ECM resulting in increased cellular adhesion, matrix mineralization and activation of MAPK-ERK pathway.

The lack of mechanical stimulus can severely impair the ability of MSCs to differentiate into osteoblasts. Studies have shown down-regulation of the focal adhesion complex, MAPK and TGF-β signaling pathways in MSCs under SMG. LIPUS is known to induce acoustic streaming in interstitial fluid and localized mechanical vibrations in the ECM [39] resulting in the local deformation of cell membranes and shear stresses and strains to osteoblasts [23,40,41,42,43,44]. These mechanical deformations subsequently activate integrin, mechanosensitive-calcium channels, G-proteins, IGF, TGF-β/BMP and gap junctions, activating different downstream pathways [18,22,23,24,25,26]. Mechanical stimulations are known to activate different signaling pathways including MAPK, beta-catenin and Akt and FAK pathway. Studies by Olkku et al. have shown ultrasound induce beta-catenin activation in osteoblast cells [27]. Furthermore LIPUS stimulation has been shown to activate Integrin/FAK/PI3K/Akt complex and MAPK-Erk pathways leading to enhances osteoblastic activity in osteoblast cells [18,25,26,45]. These studies indicate that the LIPUS treatment can activate mechanotransductive pathways and significantly increase osteogenic differentiation in stem cells. The results from this study suggest that LIPUS stimulation enhance ALP activity and collagen content to facilitate matrix mineralization. Detail mechanistic studies are required to fully understand the effects of ultrasound on MSC proliferation and differentiations.

In summary, the objective of this study was to investigate the effects of LIPUS on osteogenic differentiation of MSCs in disuse conditions. Application of LIPUS increased the expression of osteogenic genes along with increasing ALP activity and expression. LIPUS treated SMG cultures had higher collagen content in ECM and more matrix calcification. Further studies are needed to understand underlying mechanotransductive pathways and the effect of LIPUS on early osteoclastogenesis/osteogenesis in MSCs. Taken together, we conclude that LIPUS provides the essential mechanical stimulus to induce osteogenesis in a SMG environment and has the potential to be used as therapy for disuse osteoporosis due to space travel, long-term bed rest, and brain/spinal cord injuries.
